# Complete Genome Sequence of *Sulfolobus solfataricus* Strain 98/2 and Evolved Derivatives

**DOI:** 10.1128/genomeA.00549-15

**Published:** 2015-05-28

**Authors:** Samuel McCarthy, Julien Gradnigo, Tyler Johnson, Sophie Payne, Anna Lipzen, Joel Martin, Wendy Schackwitz, Etsuko Moriyama, Paul Blum

**Affiliations:** a School of Biological Sciences, University of Nebraska–Lincoln, Lincoln, Nebraska, USA; b U.S. Department of Energy-Joint Genome Institute, Walnut Creek, California, USA; c Center for Plant Science Innovation, University of Nebraska–Lincoln, Lincoln, Nebraska, USA

## Abstract

*Sulfolobus solfataricus* is a thermoacidophilic crenarcheote with a 3.0-Mb genome. Here, we report the genome sequence of *S. solfataricus* strain 98/2, along with several evolved derivatives generated through experimental microbial evolution for enhanced thermoacidophily.

## GENOME ANNOUNCEMENT

*Sulfolobus solfataricus* strain 98/2 is a thermoacidophilic chemoheterotrophic crenarcheote that grows optimally at 80°C and pH 3.0 (1). The *S. solfataricus* 98/2 genome reported in 2009 (GenBank accession no. CP001800.1, RefSeq NC_017274.1, GI: 261600703) is a deletion derivative of strain 98/2 called PBL2025 (2). It is often misconstrued as the wild-type strain 98/2, yet lacks a 50-kb region encoding numerous genes involved in sugar metabolism. A new closed and complete genome sequence for wild-type strain 98/2 referred to as SULA is presented here as GenBank accession no. CP011057 using locus tag SULA. This strain has been deposited at the Japan Collection of Microorganisms. Two additional closed and complete genomes derived from strain 98/2 are also presented and include SULB (GenBank accession no. CP011055) and SULC (GenBank accession no. CP011056). SULB and SULC resulted from extensive passage during selection for the biological trait of increased acid resistance (unpublished data).

High-molecular-weight genomic DNA was prepared from clonal cultures of the *S. solfataricus* strains as described previously (3, 4). The integrity and purity of the DNA samples were verified by spectroscopic measurements at 260/280 and 260/230 nm and confirmed by agarose gel electrophoresis. DNA and RNA library preparation was conducted using JGI’s automated process with a BioMek FX robot. The samples were sheared using a Covaris E210 sonicator followed by end repair and phosphorylation. Fragments ranging from 100 to 500 bp were selected for sequencing using an automated solid phase reversible immobilization selection system. The addition of a 3′-terminal adenine was made to the fragments, followed by adaptor sequence ligation. Genome sequencing of the libraries was done using an Illumina HiSeq 2000, generating 100-bp paired-end reads. Samples were applied to a 25-Gb 2 × 100 channel that gave 1 Gb of sequence information per sample (500× coverage). Sequences were mapped to the *Sulfolobus solfataricus* reference genome (GenBank accession no. CP001800.1; PBL2025) using Bowtie2 version 2.1.0 and SAMtools version 1.0 and corrected for the deleted region.

The fully assembled *S. solfataricus* 98/2 (SULA) genome is 2,727,337 bp in size. The genome sequence was annotated through NCBI’s online annotation pipeline and had a total of 2,924 genes, 178 pseudogenes, and 46 tRNA genes. The SULB and SULC strains had the same number of genes and tRNA genes but slightly different numbers of pseudogenes (174 and 180, respectively).

### Nucleotide sequence accession numbers. 

The *S. solfataricus* strain 98/2 (SULA) genome sequence is available at GenBank under the accession number CP011057. The evolved derivatives SULB and SULC are available under the accession numbers CP011055 and CP011056, respectively.
